# Physical Organization of DNA by Multiple Non-Specific DNA-Binding Modes of Integration Host Factor (IHF)

**DOI:** 10.1371/journal.pone.0049885

**Published:** 2012-11-14

**Authors:** Jie Lin, Hu Chen, Peter Dröge, Jie Yan

**Affiliations:** 1 Department of Physics, National University of Singapore, Singapore, Singapore; 2 Mechanobiology Institute, National University of Singapore, Singapore, Singapore; 3 Centre for Bioimaging Sciences, National University of Singapore, Singapore, Singapore; 4 Division of Molecular Genetics and Cell Biology, School of Biological Sciences, Nanyang Technological University, Singapore, Singapore; University of Oklahoma, United States of America

## Abstract

The integration host factor (IHF) is an abundant nucleoid-associated protein and an essential co-factor for phage λ site-specific recombination and gene regulation in *E. coli*. Introduction of a sharp DNA kink at specific cognate sites is critical for these functions. Interestingly, the intracellular concentration of IHF is much higher than the concentration needed for site-specific interactions, suggesting that non-specific binding of IHF to DNA plays a role in the physical organization of bacterial chromatin. However, it is unclear how non-specific DNA association contributes to DNA organization. By using a combination of single DNA manipulation and atomic force microscopy imaging methods, we show here that distinct modes of non-specific DNA binding of IHF result in complex global DNA conformations. Changes in KCl and IHF concentrations, as well as tension applied to DNA, dramatically influence the degree of DNA-bending. In addition, IHF can crosslink DNA into a highly compact DNA meshwork that is observed in the presence of magnesium at low concentration of monovalent ions and high IHF-DNA stoichiometries. Our findings provide important insights into how IHF contributes to bacterial chromatin organization, gene regulation, and biofilm formation.

## Introduction

The large chromosomal DNA (∼4.7 megabases) of Escherichia coli (*E. coli*) is a compacted structure, termed the nucleoid, with the aid of a set of nucleoid-associated proteins (NAPs) [1], [2], [3]. The nucleoid is reliably orientated and highly organized, which is crucial for important cellular processes such as gene regulation, DNA replication, and segregation of daughter chromosomes during cell divisions [4], [5]. *E. coli* cells response to various changes in environments, which often corresponds to changes in the nucleoid structure by modulating the NAPs composition. Indeed, the relative abundance of the major NAPs is found to be growth condition-specific [3], [6]. Among these NAPs, the integration host factor (IHF) is a conserved, abundant NAP expressed under various growth conditions and during different growth phases of bacteria [7]. The protein was discovered as an essential co-factor for site-specific recombination of phage λ in *E. coli* [8]. λ integrase-mediated recombination requires binding of IHF to specific DNA sequences within the phage λ attachment region where it creates sharp (>160^o^) DNA kinks upon binding [9]. IHF is also known as a transcriptional regulator that influences global gene transcription in *E. coli* [10] and *S. typhimurium* [11]. It has been suggested that gene regulation by IHF requires its DNA architectural function, which facilitates interactions between regulatory proteins and RNA polymerase [12]. IHF recognizes consensus DNA motifs consisting of small clusters of conserved nucleotide residues [13], [14], [15]. It binds to these consensus sites with high affinity [16], [17], [18]. For example, the H’ sequence that is involved in site-specific recombination [19], [20] has a dissociation constant in the range of 0.025–20 nM [16], [17], [18], [21], [22].

The intracellular concentration of IHF is rather high during all bacterial growth phases, which is somehow inconsistent with its low *K*_d_ for specific DNA binding sites. The copy number of IHF heterodimers ranges from 12000 in the exponential growth phase to 55000 in the early stationary phase, corresponding to a concentration range of 12–55 µM [6]. The high intracellular concentration range suggests that IHF may associate with DNA in a non-specific manner, and being an abundant nucleoid associated protein (NAP), contributes to bacterial chromatin organization. In addition, IHF is involved in both formation and maintenance of bacterial biofilms since it is found in complex with extracellular DNA (eDNA) within the extracellular polymeric substances (EPS) matrix of many biofilms [23], [24]. Interestingly, recent data suggest that the protein plays an important DNA architectural role in the maintenance of the eDNA meshwork [25]. However, even though non-specific DNA binding by IHF seems to be biologically important, rather little is known at the molecular level about this particular mode of binding.

Unlike IHF, its homolog protein HU, which is also a heterodimer protein and has an overall similar structure [26], has been extensively studied for its non-specific DNA binding properties. Two DNA binding modes were reported for *E. coli* HU: in high monovalent salt concentration and low protein concentration, *E. coli* HU binding leads to DNA bending similar to IHF. However, in low monovalent salt concentration and high protein concentration, *E. coli* HU can form a rigid nucleoprotein filament with double-stranded DNA [26], [27]. In addition, studies of HU from *B. stearothermophilus* (BstHU), which shares 60% sequence identity to *E. coli* HU, revealed a much stronger DNA condensation capability than *E. coli* HU. However, unlike *E. coli* HU, DNA stiffening beyond the bare DNA level was not identified for BstHU [28]. Although these studies on HU can provide some insights into the non-specific DNA binding properties of IHF, direct knowledge of non-specific IHF-DNA interactions is still lacking.

IHF is known to be able to interact with DNA both specifically and non-specifically. According to previous isothermal titration calorimetry studies, non-specific binding of IHF is favoured at low potassium concentration and high IHF-DNA stoichiometries [29], [30]. An important result from these studies is that a smaller occluded size of DNA (∼10 bp) was observed in the non-specific binding mode compared to the ∼34 bp in a specific complex. The effects of non-specific binding of IHF on the mechanical properties of DNA have been studied recently in single-DNA stretching experiments using λ-DNA [31], which contains only four consensus IHF sites [19]. It was found that the addition of IHF only moderately reduced DNA extension at the saturation binding concentration of IHF [31]. In these studies, the effect of IHF binding on the force response of DNA is similar to that predicted for DNA bending proteins [32], [33], suggesting that non-specific binding of IHF also alters DNA structure. It appears that at saturation binding, less DNA bending than expected from the specific binding of IHF is observed [32], [33]. This suggests that non-specific binding of IHF introduces weaker DNA bending under the conditions tested or that it can introduce sharp DNA bending but only sparsely binds to DNA even at saturation binding. Additionally, a recent study suggests a non-specific conformational capture step, in which thermal fluctuations in DNA adopt “pre-bent” conformations that can be subsequently captured and stabilized by IHF. This conformational capture of pre-bent DNA conformations is proposed to be crucial for sequence recognition by IHF [34]. Such a model is, therefore, consistent with the existence of DNA bending conformations in non-specific IHF-DNA complexes.

Little is known about the dependences of the non-specific DNA binding of IHF on physiological factors such as IHF concentration, monovalent and divalent salt concentrations, pH, temperature, and molecular crowding. However, such knowledge is crucial to understand the responses of the *E. coli* nucleiod to these frequently changing factors, which has been highlighted from recent studies of several other bacterial NAPs, such as *E. coli* H-NS and StpA and *P.aeruginosa* MvaT, in which these NAPs can sense environmental changes and adapt their DNA binding properties accordingly [35], [36], [37].

In this study, we addressed these questions and investigated non-specific interactions between IHF and DNA using magnetic tweezers and atomic force microscopy (AFM). Our results uncovered multiple DNA binding modes of IHF which result in complex DNA structures. These binding modes are controlled by conditions such as protein, monovalent salt, and magnesium concentrations. Our results have important implications for global gene regulation, bacterial nucleoid organization, and biofilm formation/maintenance.

## Materials and Methods

### Proteins

Purified *E. coli* wild-type IHF was a kind gift of D. Esposito to P.D., which was expressed and purified according to the original protocol from Howard Nash [38].

### Transverse Magnetic Tweezers Measurements

Biotin labeled λ-DNA (48502 bp, New England Biolabs) molecules at the two DNA ends of the opposite DNA strands were used for single-DNA stretching experiments. DNA stretching was performed using a transverse magnetic tweezers setup, which can stretch the DNA in the focal plane [39]. One end of DNA was attached to a streptavidin-coated cover glass edge, and the other end was attached to a 2.8-µm paramagnetic bead (Dynalbeads M-280, Invitrogen, Singapore). The DNA is immersed in a flow channel, in which the buffer solution can be changed. A pair of permanent magnets is used to apply force on the tethered paramagnetic beads. A 40 X microscope objective is used to image the tethered bead onto a CCD camera (Pike F-032, Allied Vision Technologies, Germany) at ∼100 frames per second. A home-written software with LabVIEW (National Instruments, US) was used to track the paramagnetic bead. The DNA extension is determined from the centroid of the bead to the edge of the cover glass. The stretching force by the magnet is calculated by the bead thermal motion [40]:
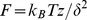
where 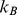 is the Boltzmann constant, 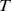 is the temperature, 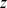 is the measured extension of the DNA, and 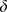 is the variance of bead fluctuation in a direction perpendicular to the stretching force.

To make sure that the stretched DNA is a single tether, the measured DNA force-extension curve is fitted with the Marko-Siggia formula [41] in the force range from 0.1 pN to 10 pN. DNA is determined to be a single tether if the persistence length is fitted to be A≈50±5 nm.

### Atomic Force Microscopy (AFM) Imaging

All imaging was done on glutaraldehyde-coated mica surface, which was prepared according to ref. 44 [42]. Briefly, 50 µl of 0.1 % (v/v) (3-aminopropyl)-triethoxysilane (APTES) solution diluted with deionised water is incubated for 10 minutes on freshly cleaved mica which is subsequently rinsed extensively with deionised water and dried with nitrogen gas. Following the step, 50 µl of 1 % (v/v) glutaraldehyde solution is incubated for 15 minutes on the APTES-modified mica which is again subsequently rinsed and dried before use. Such glutaraldehyde-modified surface was able to immobilize DNA-protein complexes by crosslinking the amine groups of the proteins bound to the DNA to the surface. As the glutaraldehyde molecules are covalently bound to the surface, they do not diffuse into the solution and therefore do not non-specifically aggregate proteins or DNA-protein complexes. Such surface has been shown less perturbing the stability of DNA-protein interactions and is friendly to DNA-protein complex imaging [43]. As immobilization of DNA-protein complexes on the glutaraldehyde-modified surface does not depend on the presence of magnesium, the effects of magnesium on the conformations of DNA-protein complexes can be studied [35], [36], [37], [44].

The DNA substrate used for the imaging experiments is 5386 bp Φ×174 dsDNA RF1 (New England Biolabs) linearized by PstI (New England Biolabs). DNA of fixed concentration (0.2 ng/ul) was incubated with different IHF concentrations and in different solutions with 10 mM Tris (pH 7.4). DNA was incubated with IHF for 45 minutes and transferred to mica for additional 20 minutes before imaging in air. Imaging was performed using Molecular Imaging 5500 AFM (Molecular Imaging, Agilent Technologies) on acoustic AC mode. Silicon cantilevers (Photonitech, Singapore) with a resonant frequency of ∼300 kHz and force constant of 40 N/m were used. Gwyddion software (http://gwyddion.net/) was used to process all the images.

## Results

### KCl Dependency of the Influence of IHF on the DNA Force Response

**Figure 1:**
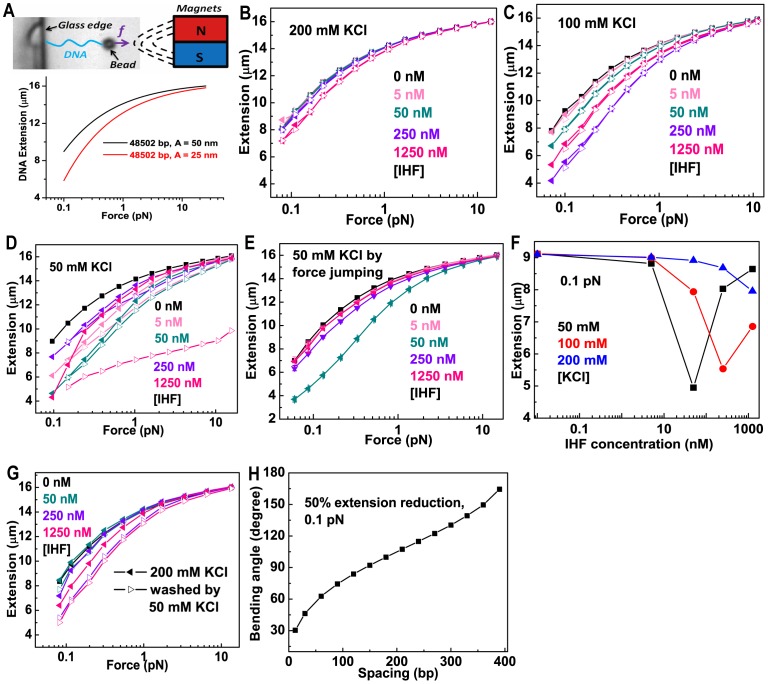
Influences of IHF on DNA force response in the absence of magnesium. (A) Top-panel: Schematic diagram of the transverse magnetic tweezers setup used in this paper. Bottom panel: force-extension curves of λ-DNA according to the Marko-Siggia formula for the protein-free DNA persistence length of 50 nm (black) and a reduced persistence length of 25 nm (red). (B–D) Effects of IHF on the force response of λ-DNA at varying concentrations of KCl and pH 7.4. Force-extension curves of DNA in the force-decreasing (filled triangles) and force-increasing (open triangles) scans at the indicated concentrations of IHF in 200 mM KCl (B), 100 mM KCl (C), and 50 mM KCl (D), respectively. (E) Force-extension curves measured in 50 mM KCl by force jumping. (F) DNA extension as a function of the IHF concentration at 0.1 pN at different KCl concentrations. Data at 0.1 pN were obtained from the force-extension curves at corresponding KCl concentrations in Figure 1B–C and 1E by linear interpolation using two nearest neighbouring data points adjacent to 0.1 pN. (G) Decreasing KCl concentration from 200 mM to 50 mM drives a switch from smaller to higher degrees of DNA bending. Filled triangles represent force-extension curves of DNA incubated in 200 mM KCl at the indicated concentration of IHF. Open triangles represent force-extension curves of DNA after lowering the KCl concentration to 50 mM and removing IHF. (H) The DNA bending angle as a function of the spacing of IHF bound to DNA that causes 50% reduction in DNA extension at 0.1 pN.

To determine how IHF binds to DNA, we studied the mechanical response of a single λ-DNA molecule (48502 bp) to IHF-binding using a transverse magnetic tweezers setup (Figure 1A). Theoretical predictions revealed that binding of DNA-distorting proteins can change the force-extension curves of DNA, hence providing information on the binding mechanism [32]. The binding of DNA-bending protein results in a lowered apparent DNA bending persistence length, causing shortening in DNA extension at small forces as illustrated in Figure 1A. Note the four consensus IHF sites on the λ-DNA [19] will not affect the DNA force response, as the number of specific bends is too small to cause detectable influence on the force response of the 48502 bp λ-DNA [32], [45].

The force-responses of single λ-DNA molecules were studied in response to changes both in IHF and KCl concentrations at 20°C and pH 7.4. For IHF concentrations ranging from 0–1250 nM, the force-extension curves were recorded in 200 mM KCl (Figure 1B). To determine if IHF binding reached a steady or equilibrium state, the data were recorded using a force-decreasing scan, during which the force was sequentially decreased from higher to lower values, followed by a force-increasing scan through the same set of force values. If protein binding and unbinding are fast and reach equilibrium over the experimental time scale, the force-extension curve obtained in the force-increasing scan should overlap with that obtained in the force-decreasing scan. Otherwise, the force-extension curve in the force-increasing scan should be lower than that in the force-decreasing scan due to DNA extension reduction caused by protein-induced DNA bending or DNA folding (i.e., hysteresis in force-extension curve). At each force, data were recorded for 30 s, and the data obtained in the final 5 s were averaged to calculate the extension. At 200 mM KCl, no hysteresis was observed. At IHF concentration below 250 nM, the DNA extension almost overlaps with that of the naked DNA without protein. At 1250 nM IHF, which exceeds the saturation binding concentration of IHF, ∼500 nM (the force-extension curves remain unchanged at >500 nM IHF) [31], IHF binding weakly reduces the DNA extension: the DNA becomes ∼20% shorter than naked DNA at ∼0.1 pN. Overall, our data are consistent with previous results obtained under the same conditions [31].

We next measured how IHF-DNA interactions are influenced by various KCl concentrations. We found that in the presence of IHF, DNA is significantly less extended at 100 mM KCl than at 200 mM KCl (Figure 1B–C). Extension at 50 nM IHF in 100 mM KCl (Figure 1C) is comparable to that obtained at 1250 nM IHF in 200 mM KCl, where saturation binding is achieved (Figure 1B). If IHF induces equal degrees of DNA bending at 200 mM KCl and 100 mM KCl, saturation binding should occur at 100 mM KCl and ∼50 nM IHF. However, when IHF concentration is increased above 50 nM, DNA extension decreases. This suggests that IHF reduces DNA extension through different mechanisms at 100 mM KCl and 200 mM KCl. Because no hysteresis was observed between the force-decreasing and force-increasing scans, this increased DNA extension reduction is not likely due to higher order DNA condensation caused by mechanisms such as DNA looping or DNA bridging. Rather, it may be due to sharper DNA bending than that occurring at 200 mM KCl. In addition, DNA extension was non-monotonically dependent on IHF concentration at 100 mM KCl. When IHF concentration was increased from 250 nM to 1250 nM in 100 mM KCl, DNA extension increased.

To further illuminate how KCl affects DNA-binding properties of IHF, we repeated this experiment at 50 mM KCl. Similar non-monotonic dependence of DNA bending on IHF concentration was observed with maximal bending occurring at ∼50 nM IHF (Figure 1D). Note that in 50 mM KCl, slow hysteresis between the force-decreasing and force-increasing stretching curves occurred. Such slow DNA folding signal can be filtered out by a quick force jumping method explained in Supporting Information (Methods S1A and Figure S1). The force extension curve obtained by force jumping (Figure 1E), resembles results obtained in 100 mM KCl (Figure 1C) in terms of the maximal DNA extension reduction and the non-monotonic dependence of DNA extension on IHF concentration. Therefore, faster IHF-induced DNA bending in 50 mM KCl probed by the force-jumping method seems to be of the same nature as DNA bending in 100 mM KCl. The hysteresis observed in 50 mM KCl therefore indicates a different DNA folding mechanism from bending in 100 mM KCl. It indicates either an even sharper degree of DNA bending with slower kinetics, or DNA condensation into higher order complex structures by IHF.

The non-monotonic dependence of DNA extension on IHF concentration in 100 mM or 50 mM KCl probed by force jumping suggests that the level of DNA bending is mediated by IHF concentration and that sharper DNA bending is not favoured at higher IHF concentrations. To quantify this phenomenon, DNA extensions recorded at 200 mM KCl (Figure 1B), 100 mM KCl (Figure 1C), and 50 mM KCl by force jumping (Figure 1E) are plotted as functions of IHF concentration at the same force of 0.1 pN (Figure 1F). At 200 mM KCl, DNA extension monotonically decreases as IHF concentration increases, whereas at 100 mM and 50 mM KCl, there appears to be a critical IHF concentration below which DNA extension monotonically decreases as IHF concentration increases and above which DNA extension monotonically increases as IHF concentration increases. These results reveal complex non-specific interactions between IHF and DNA. Binding of IHF to DNA, inducing a fixed bending angle, cannot explain these results. The existence of at least two non-specific DNA bending states that depend on both KCl and IHF concentration would explain the differential force-response of the DNA-IHF complex to these factors.

### KCl Controls the Degree of Bending in IHF-DNA Complexes

At an IHF concentration where saturated DNA binding is observed (e.g. 1250 nM), DNA is more extended in 200 mM KCl than the shortest DNA extension at 50 mM or 100 mM KCl (Figure 1F). To determine if decreasing the KCl concentration induces increased DNA bending, a DNA tether was incubated at varying concentrations of IHF in 200 mM KCl and then at 50 mM KCl without free IHF proteins. If a sufficient amount of IHF remains associated with DNA, one should expect to see the response of IHF-DNA complexes to the change in KCl concentration undisturbed by free IHF in solution.

At 200 mM KCl and an IHF concentration of 50 nM IHF, the force-extension curve almost overlaps with the reference curve obtained from naked DNA before IHF was added (Figure 1G). However, at 50 mM KCl in the absence of IHF, DNA extension was reduced slightly by ∼600 nm at ∼0.08 pN (Figure 1G). This decrease in DNA extension was not caused by effects of salt on the elasticity of naked DNA, as the force-response of DNA is almost identical in KCl concentrations ranging from 50–200 mM (Figure S2). Repeating this experiment at IHF concentration of 250 nM or the saturating concentration of 1250 nM, we obtained similar results but with greater DNA extension reduction (Figure 1G). These findings support the existence of at least two distinct DNA bending modes of the IHF-DNA complex. Because there was no free IHF in the 50 mM KCl solution, the reduced extension that occurred after changing the buffer should have resulted from the response of DNA-bound IHF to the change in KCl concentration.

### The Degree of DNA Bending at Non-specific DNA Sites is Substantially Smaller than that at Specific Cognate Sites

Mainly three factors influence the force-extension curves: DNA bending rigidity, the degree of bending introduced by IHF, and the occupancy of DNA by IHF. The DNA bending rigidity is characterized by the DNA persistence length, which was measured to be ∼50 nm [41], [46], leaving the DNA bending angle and IHF occupancy two undetermined factors that control the shape of the force-extension curves. The force-extension curves in Figure 1 indicate two DNA bending states influenced by the concentration of KCl. An interesting question is whether increased bending in 100 mM KCl and 50 mM KCl is comparable to the ∼160^o^ kink observed in the specific IHF-H’ complex [9].

According to Figure 1F, at 0.1 pN, the extension of DNA decreased the most at IHF concentrations of 250 nM in 100 mM KCl and 50 nM in 50 mM KCl, where DNA extension was shortened by ∼50% from the naked DNA at the same force. To compare with experimental data, we simulated the kink bending angle as a function of the occupancy density of IHF (number of base pairs between adjacent IHF) that can decrease extension by 50% at 0.1 pN DNA (Figure 1H and Methods S1B) over a wide range, i.e. from 1 IHF per 390 bp to 1 IHF per 12 bp. As shown in Figure 1H, for bending of 160^o^, low protein occupancy density around one IHF per 390 bp is able to reduce extension by 50%. However, in our experiments, the greatest decrease in extension occurred at critical concentrations of IHF above which overcrowding of IHF occurs. Therefore, the IHF occupancy density is expected to be higher and as a result the degree of bending is expected to be smaller, in order to explain the results. For example, if we assume an occupancy density of 1 IHF per 34 bp (the size of fully wrapped DNA in a specific IHF-H’ complex), a bending angle of ∼50^o^ could explain the result. Although the actual IHF occupancy density in our experiments was not determined, these data suggest that in the non-specific DNA binding mode, the extent of IHF-induced DNA bending in 100 mM KCl and 50 mM KCl is likely much smaller than, for example, that determined with the specific binding to the H’ site. This result is consistent with a previous study that reported a smaller occluded size of DNA (∼10 bp) in the non-specific binding mode than the ∼34 bp in a specific IHF-DNA complex. This implies that shorter DNA segment is wrapped around an IHF heterodimer [29]. It is also in agreement with an earlier study based on fluorescence resonance energy transfer that reported less DNA bending in a non-specific DNA-IHF complex [47].

### IHF Induces More Compact DNA Conformations at Low KCl Concentration

**Figure 2:**
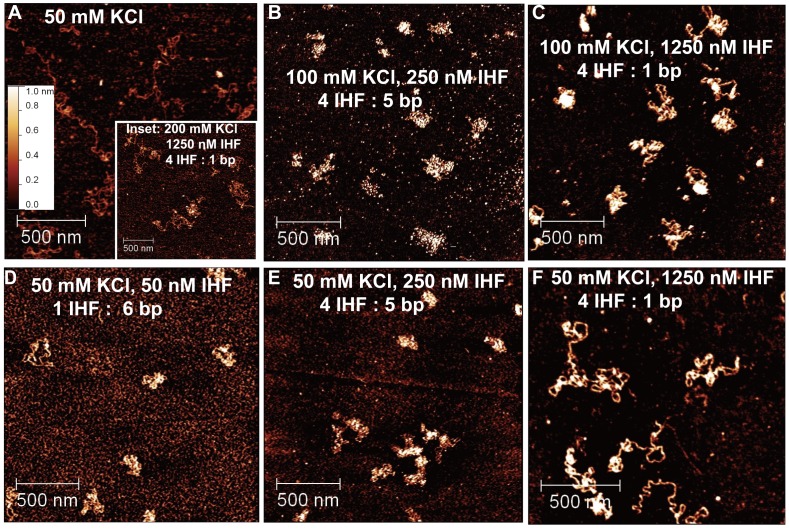
AFM analysis of linearized double-stranded Φx174 DNA incubated with varying concentrations of IHF. IHF heterodimer to DNA base pair ratio is indicated in each image panel. (A) Naked DNA that was not incubated with IHF in 50 mM KCl. Similar DNA conformation was found in 200 mM KCl with 1250 nM IHF, which is the highest protein concentration (Inset figure). (B–C) DNA molecules incubated in 100 mM KCl with 250 nM IHF (B) and 1250 nM IHF (C) respectively. (D-F) DNA molecules incubated in 50 mM KCl with 50 nM IHF (D), 250 nM IHF (E) and 1250 nM IHF (F), respectively.

In order to obtain more information of the DNA organization triggered by IHF at different KCl concentrations, we performed AFM imaging experiments on glutaraldehyde-coated mica surfaces that are particularly useful for imaging DNA-protein complexes [42], [43]. As the glutaraldehyde molecules are covalently bound to the surface, they do not diffuse into the solution and therefore do not non-specifically crosslink proteins or DNA-protein complexes. Such surface has been shown to be less perturbing to the stability of DNA-protein interactions. At 50 mM KCl or 200 mM KCl, protein-free linear dsDNA (Φ×174, 5386 bp), which does not contain any consensus IHF sites, assumed extended random coiled conformations (Figure 2A), which are similar to conformations of DNA on APTES-coated mica surfaces (Figure S3A) but are more compact than DNA on a freshly cleaved mica surface containing magnesium ions (Figure S3B). In 200 mM KCl, addition of IHF up to the highest concentration (1250 nM) did not show an apparent influence on DNA conformations when compared to protein-free DNA (inset of Figure 1A). This observation is consistent with the single-DNA stretching experiments where DNA extension was only moderately reduced in 200 mM KCl (Fig. 1B and 1F). In contrast, in 100 mM KCl, addition of 250–1250 nM IHF induced more compact DNA conformations (Figure 2B–C). At a lower KCl concentration of 50 mM, addition of 50–250 nM IHF induced similar compacted DNA conformations (Figure 2D–E) to those in 100 mM KCl (Figure 2B–C). However, in 50 mM KCl and 1250 nM IHF (Figure 2F), DNA became significantly more extended than in 50 and 250 nM IHF.

In general, these AFM imaging results are consistent with the results from single-DNA stretching experiments: 1) DNA is more sharply bent in 100 mM and 50 mM KCl than in 200 mM KCl, and 2) in low salt, the DNA bending angle non-monotonically depends on the concentration of IHF, as demonstrated in Figure 2F. In addition, we did not find apparent evidence that DNA can be condensed into higher order structures in 50 mM KCl. Such DNA condensation mechanism would predict DNA-protein complexes of varying sizes expected from inter-DNA aggregations mediated by IHF; however, the size of the DNA-IHF complexes identified by AFM do not vary a lot.

### IHF Condenses DNA into Higher Order Structures in the Presence of Magnesium

We next investigated the influence of magnesium on DNA organization by non-specific IHF binding. Magnesium is known to be essential for many enzymatic reactions in bacteria and is present in bacteria at concentrations up to 4 mM [48]. It is also critical for chromosomal condensation and DNA repair [48], [49]. Recent experiments suggest that magnesium is also important for regulating DNA binding properties of bacterial NAPs, such as H-NS and StpA [35], [36], [50], [51]. Hence, it will be interesting to ask how magnesium affects binding of other NAPs to DNA.

**Figure 3:**
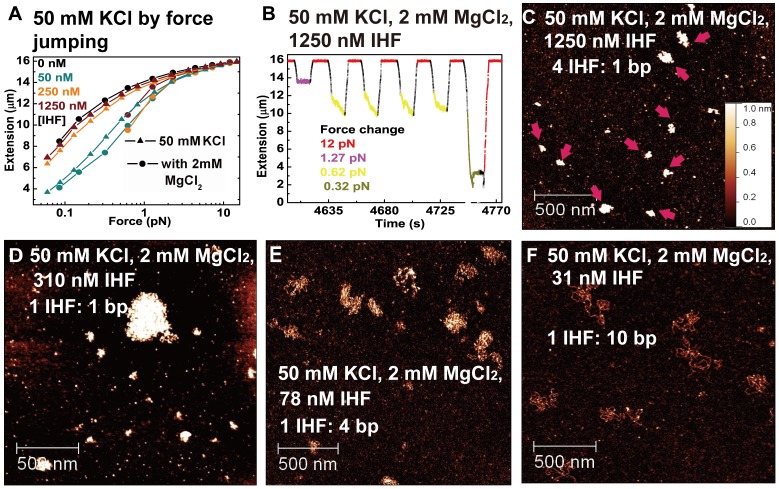
Effects of magnesium on DNA condensation in the presence of IHF. (A) Force-extension curves obtained by force jumping. Triangles and circles represent data obtained in the absence and presence of 2 mM MgCl_2_, respectively. For 250 nM and 1250 nM IHF, data are not shown for force <0.6 pN because DNA extension was below the minimal extension (∼2 µm) that could be measured by our instrument. (B) DNA folding time course at various values of lower force and unfolding time course at the high force of ∼12 pN in 1250 nM IHF. (C–F) Atomic force microscopy analysis of DNA molecules incubated in 50 mM KCl and 2 mM MgCl_2_ with 1250 nM IHF (C), 310 nM IHF (D), 78 nM IHF (E) and 31 nM IHF (F). IHF heterodimer to DNA base pair ratio is indicated in each image panel.

Single-DNA stretching experiments were performed first to investigate the effects of magnesium on binding of IHF to DNA. In 200 mM KCl, we found that addition of 2 mM MgCl_2_ did not affect IHF-binding. The resulting force-extension curves in 0 – 1250 nM IHF are similar to that in 200 mM KCl without magnesium (Figure S4A). We next used KCl concentration of 50 mM where sharper DNA bending and DNA condensation are detected. Using force jumping, we first measured the DNA force-extension curves in the absence of magnesium as controls (these data already appeared in Figure 1E and Figure 1F). Then, in 50 nM IHF and 2 mM MgCl_2_, the DNA extension is comparable to that obtained in the absence of MgCl_2_ at >2 pN but slightly shorter at <1 pN. In 250 nM and 1250 nM IHF and 2 mM MgCl_2_, DNA extension became significantly shorter than that obtained in the absence of magnesium at ∼0.6 pN (Figure 3A). Data points below 0.6 pN are not shown, because DNA extension was reduced to below 2 µm within 10 s at these force values, which is too short to be measured by our magnetic tweezers setup due to the shadow of the cover glass edge indicated by the left-hand arrow in Figure 1A. To observe DNA extension reduction more clearly, the DNA folding time courses at ∼0.6 pN and ∼0.3 pN are shown before DNA extension was reduced below 2 µm (Figure 3B). The DNA folding speed is fast at low force, exceeding 1 µm/s extension reduction at ∼0.3 pN. For comparison, folding in the absence of magnesium is much slower even at the lowest force of ∼0.1 pN (Figure S5).

In order to understand whether magnesium-dependent DNA folding is caused by DNA condensation into higher order structures, or by sharper DNA bending, we performed AFM imaging to visualize the DNA-IHF complexes. In these experiments, DNA concentration was fixed at 0.2 ng/µl (base pair molar concentration ∼310 nM). At 1250 nM IHF and 50 mM KCl (Figure 3C), the IHF-DNA complex was more compact in the presence of magnesium than in its absence (Figure 2F). Importantly, the size of these highly compact DNA-IHF complexes is heterogeneous, suggesting that different amounts of DNA are packaged inside each complex, as indicated by arrows in Figure 3C. Dilution of IHF to 310 nM (Figure 3D) and 78 nM (Figure 3E) reduces the level of DNA compaction. At 31 nM IHF (1 IHF dimer: 10 bp), DNA compaction is not observed. For comparison, in the presence of 200 mM KCl, where only weak DNA bending is observed in single-DNA stretching experiments, AFM imaging at 1250 nM IHF consistently shows that DNA assumes coiled conformations similar to naked DNA (Figure S4B).

These results indicate that magnesium promotes higher order DNA compaction at ∼50 mM KCl, sufficiently high IHF concentrations (∼250 nM or higher), and high IHF: DNA stoichiometries (∼1 IHF:4 bp or higher). Considering that IHF is an abundant NAP and magnesium exists *in vivo* in the mM range, these findings imply that the non-specific binding of IHF to bacterial DNA could be important for bacterial DNA compaction. In addition, it may also be important for the organization of eDNA in biofilms, although the exogenous concentration of MgCl_2_ is likely be different in different environments.

## Discussion

Our study revealed that the interaction between IHF and DNA is complex, with IHF binding to DNA via different modes that induce different DNA bending patterns. Furthermore, these different DNA binding modes are sensitive to environmental factors such as KCl, magnesium, and force. High concentrations of KCl induce weak DNA bending, and a saturated concentration of IHF does not condense DNA further. At <100 mM KCl and unsaturating IHF levels, sharper DNA bending occurs resulting in DNA extension reduction. This state of increased DNA bending is inhibited at higher IHF concentrations, which leads to the non-monotonic relation between DNA extension and IHF concentration. This less bent DNA conformation is energetically favourable at high concentrations of IHF, because it will likely make more DNA available to accommodate more IHF proteins. Moreover, a physiological concentration of magnesium enhanced DNA compaction, suggesting a possible role of non-specific DNA binding by IHF in the packaging of bacterial DNA. In cells, other multivalent cations or polyamines also exist which may directly [52] or in cooperation with proteins [53] condense DNA. Therefore, it will be interesting to investigate how they influence the DNA organization by IHF in future studies.

**Figure 4:**
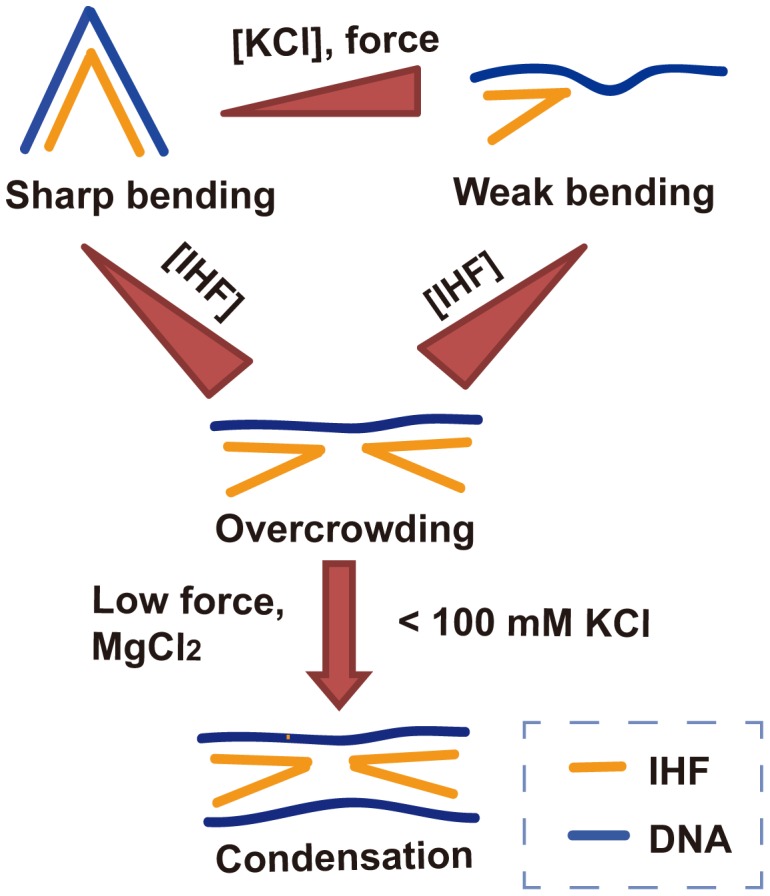
Schematic model of IHF-DNA interaction. The conformational states of the DNA-IHF complex and their dependence on force, [IHF], [KCl] and [MgCl_2_] are summarized here. Yellow represents an IHF dimer, and blue represents dsDNA. Dark red right trangles indicate increasing values of force and [KCl].

These DNA binding modes, their dependence on environmental factors, and the resulting DNA deformations and organizations are summarized in Figure 4: 1) when binding of IHF is not saturated, weaker and sharper bending conformations are regulated by the concentration of KCl or tension; 2) at high concentrations of IHF, DNA always adopts the weaker bending conformation regardless of the KCl concentration and tension due to overcrowding of IHF on DNA; 3) when overcrowding occurs at low concentrations of KCl, the exposed IHF DNA binding interface can also interact with other DNA segments or molecules, leading to further higher-order DNA condensation in the presence of magnesium in the mM range. Note that Figure 4 suggests that the sharper bending mode occupies more DNA than the weaker bending mode, as a less bent conformation in general means a less wrapped DNA state. This is also consistent with a smaller occluded size of DNA (∼10 bp) in the non-specific binding mode than the ∼34 bp occluded size in the sharply bent specific binding mode of IHF [29], [30].

### Comparison with Other Non-specific DNA Folding Proteins

DNA bending and higher order DNA condensation represent two commonly observed DNA folding mechanisms utilized by DNA architectural proteins. Several well known DNA bending proteins such as HU and Fis in *E. coli*, and HMGB1 and NHP6A in eukaryotic cells, have been investigated in single-DNA stretching and/or AFM imaging experiments [26], [54], [55]. Among these proteins, it will be particularly interesting to compare the results obtained for IHF in this study with previous studies of its cousin HU, which has an overall similar structure [26]. In contrast to the DNA stiffening effects of *E.coli* HU in low monovalent salt concentration and high protein concentration [26], [27], DNA stiffening beyond bare DNA level was not identified for IHF in all conditions explored in our studies. In the presence of magnesium and high IHF concentration, IHF can organize DNA into higher order complexes (Figure 3), which was also not found in previous studies of *E. coli* HU. However, several DNA binding features of IHF revealed in our studies are similar to those reported for BstHU, including the non-monotonic dependence of DNA force-extension curves on the protein concentration, and the lack of DNA stiffening beyond the bare DNA level at very high protein concentrations [28]. It will also be interesting to compare with other NAPs in *E. coli* that can organize DNA into higher order structures. Fis and Dps are known to be able to crosslink DNA into higher order DNA complexes [56], [57], [58]. Unlike IHF, however, DNA condensation by these two NAPs does not require the presence of magnesium. As such, IHF is an NAP with multiple DNA binding modes which are in many aspects distinct from other DNA folding NAPs.

### Implications on Global Bacterial Gene Regulation

IHF influences global transcription in *E. coli* [10] and *S. typhimurium* [11]. It has been suggested that IHF positively regulates gene transcription by bending DNA to facilitate contact between regulatory proteins and RNA polymerase [12]. Our finding that IHF induces more than one state of DNA bending mediated by several factors suggests that global gene regulation by IHF may be influenced by physiological factors that control DNA bending. However, regulation of specific genes by IHF remains most likely controlled by high-affinity binding of IHF to specific DNA sequences, and the DNA conformations induced by these specific interactions may differ from those induced by non-specific interactions.

### Implications on Packaging of Chromosomal DNA in Bacteria

IHF is the second most abundant NAP in the early stationery phase with a copy number of ∼55000 and a concentration of ∼55 µM [1]. Interestingly, the nucleoid of *E. coli* becomes more compact when it enters the stationary phase [51]. Because Dps is the most abundant NAP in the early stationery phase and it condenses DNA, it is believed to be responsible for the packaging chromosomal DNA in bacteria [58]. Our results suggest that IHF may also play a major role in DNA compaction during the early stationary phase, because it condenses DNA at physiological concentrations of magnesium.

We also want to point out that, *in vivo*, there are many other abundant nucleoid associated proteins (NAPs) that will compete with IHF. The total concentration of NAPs may well exceed 300 µM [1]. The average NAP to DNA ratio *in vivo* will then become greater than 1 protein: 10 bp, which is comparable to our AFM imaging at 1∶10–1∶1 (protein to base pair ratio) range. Moreover, molecular crowding effects may also play a role in enhancing DNA condensation *in vivo* [59]. In our single-DNA stretching experiments, the ratio was not controlled due to the nature of single-DNA stretching experiments where only one DNA molecule is stretched. In all single-DNA stretching experiments, the IHF to DNA ratio is always in excess. In such experiments, only the concentration of the protein is meaningful.

In a recent super-resolution imaging experiment, the intracellular localization of several NAPs including IHF was imaged. IHF was found to form small clusters widely spread on the *E. coli* chromosome [60]. The cause of the IHF clustering may be due to sequence preference of IHF or a result from competition with other NAPs binding to chromosomal DNA. Additional studies are needed to elucidate where IHF localizes on *E. coli* chromosomal DNA and how it contributes to DNA packaging in the presence of other NAPs.

### Implications on Biofilm Maintenance

Bacteria can form an organized, functional, and complex community called a biofilm. It has been estimated that most bacterial infections involve biofilm formation during disease progression [25], [61]. EPS that contain polysaccharides, proteins, nucleic acids, and lipids are critical to the formation and maintenance of biofilms [61]. The EPS provide the scaffold for the three-dimensional architecture of the biofilm and protect the bacteria within the biofilm [61].

eDNA is a common component of the EPS and it has been shown that the eDNA meshwork plays an important role in stabilizing the biofilms [23]. Interestingly, IHF and HU have been found in the eDNA meshwork and they localize to kinked DNA and crossed DNA [25], [62]. Importantly, removal of these proteins leads to biofilm disassembly or biofilm debulking [25]. These results are supported by our finding that IHF can bend DNA and, in the presence of MgCl_2_, condense DNA into a meshwork-like structure. Further, it was reported that interaction of multivalent inorganic ions with EPS can greatly influence the mechanical stability of biofilms [61]. For example, the presence of divalent ion increased the mechanical stability of mucoid *P. aeruginosa* biofilms [63]. Although this effect was previously explained by the divalent ion-mediated crosslinking of polyanionic alginate molecules, our result of the effects of magnesium suggests that divalent ion-enhanced, IHF-induced DNA crosslinking occurs in the eDNA meshwork. Therefore, our results also provide insights into the structural roles of IHF in supporting biofilm integrity.

### Conclusion

In summary, we have shown that distinct modes of non-specific binding of IHF to DNA result in complex DNA conformations. Changes in KCl concentration, IHF concentration, and force can change the degree of DNA bending. In addition, IHF can crosslink DNA into a highly compact meshwork structure that is enhanced by magnesium. Our findings provide new insights into the interactions and functions of IHF in bacterial gene regulation, chromosome packaging, and biofilm maintenance.

## Supporting Information

Figure S1 **λ-DNA extension time-course in a force-jumping experiment at 50 nM IHF in 50 mM KCl.** Black indicates the highest force (14.7 pN). Forces of lower values are indicated by different colors.
(TIF)

Figure S2 **Force-extension curves of λ-DNA in 50–200 mM KCl and pH 7.4 (10 mM Tris).** These show that the force-response of DNA is almost identical in the whole KCl concentration range.
(TIF)

Figure S3 **AFM imaging of naked Φx174 DNA on APTES-coated mica and freshly cleaved mica.** (A) Naked DNA in 200 mM KCl on APTES-modified mica. (B) Naked DNA in 10 mM MgCl_2_ (divalent salt bridging) on Fresh-mica surface.
(TIF)

Figure S4 **IHF-DNA interaction in 200 mM KCl in the presence of magnesium.** (A) Effects of magnesium on DNA conformations in 200 mM KCl. Force-extension curves in force-decreasing and force-increasing scans of λ-DNA at the indicated IHF concentrations, which are similar to those obtained in 200 mM KCl in the absence of magnesium (Figure 1B). (B) AFM imaging of DNA molecules complexed with 1250 nM IHF in 200 mM KCl in the present of 2 mM MgCl_2_.
(TIF)

Figure S5 F**olding time course of λ-DNA with 1250 nM IHF in 50 mM KCl solution.** The compaction without magnesium is much slower (blue curve), even at the lowest force ∼0.07 pN, compared to that in the similar 50 mM KCl solution with magnesium (Figure 3B). Moreover, the compaction is not as stable as that with magnesium, as it can be easily unfolded under at ∼8.7 pN (red curve). The green dot grids are used as a comparison criterion for the DNA extension reduction.
(TIF)

Methods S1 **Supplementary Methods.** (A) Quick force jumping method (B) Simulation details.
(DOC)

## References

[1] Azam TA, Ishihama A (1999) Twelve species of the nucleoid-associated protein from Escherichia coli. Sequence recognition specificity and DNA binding affinity. J Biol Chem 274: 33105–33113. 10551881 10.1074/jbc.274.46.33105

[2] Ishihama A (2009) The nucleoid: an overview. EcoSal – Escherichia coli and Salmonella: Cellular and Molecular Biology; Boek A CRI, Kaper JB, Karp PD, Neidhardt FC, Nystrom T, Slauch JM, Squires CL & Ussery D, editor: ASM Press, Washington, DC.

[3] Stavans J, Oppenheim A (2006) DNA-protein interactions and bacterial chromosome architecture. Phys Biol 3: R1–10. 17200598 10.1088/1478-3975/3/4/R01

[4] Dame RT (2005) The role of nucleoid-associated proteins in the organization and compaction of bacterial chromatin. Mol Microbiol 56: 858–870. 15853876 10.1111/j.1365-2958.2005.04598.x

[5] Toro E, Shapiro L (2010) Bacterial chromosome organization and segregation. Cold Spring Harb Perspect Biol 2: a000349. 20182613 10.1101/cshperspect.a000349 PMC2828278

[6] Ali Azam T, Iwata A, Nishimura A, Ueda S, Ishihama A (1999) Growth phase-dependent variation in protein composition of the Escherichia coli nucleoid. J Bacteriol 181: 6361–6370. 10515926 10.1128/jb.181.20.6361-6370.1999 PMC103771

[7] Ishihama A (2010) Prokaryotic genome regulation: multifactor promoters, multitarget regulators and hierarchic networks. FEMS Microbiol Rev 34: 628–645. 20491932 10.1111/j.1574-6976.2010.00227.x

[8] Nash HA, Robertson CA (1981) Purification and properties of the Escherichia coli protein factor required for lambda integrative recombination. J Biol Chem 256: 9246–9253. 6267068

[9] Rice PA, Yang S, Mizuuchi K, Nash HA (1996) Crystal structure of an IHF-DNA complex: a protein-induced DNA U-turn. Cell 87: 1295–1306. 8980235 10.1016/s0092-8674(00)81824-3

[10] Arfin SM, Long AD, Ito ET, Tolleri L, Riehle MM, et al (2000) Global gene expression profiling in Escherichia coli K12. The effects of integration host factor. J Biol Chem 275: 29672–29684. 10871608 10.1074/jbc.M002247200

[11] Mangan MW, Lucchini S, Danino V, Croinin TO, Hinton JC, et al (2006) The integration host factor (IHF) integrates stationary-phase and virulence gene expression in Salmonella enterica serovar Typhimurium. Molecular Microbiology 59: 1831–1847. 16553887 10.1111/j.1365-2958.2006.05062.x

[12] Santero E, Hoover TR, North AK, Berger DK, Porter SC, et al (1992) Role of integration host factor in stimulating transcription from the sigma 54-dependent nifH promoter. J Mol Biol 227: 602–620. 1404379 10.1016/0022-2836(92)90211-2

[13] Goodrich JA, Schwartz ML, McClure WR (1990) Searching for and predicting the activity of sites for DNA binding proteins: compilation and analysis of the binding sites for Escherichia coli integration host factor (IHF). Nucleic Acids Res 18: 4993–5000. 2205834 10.1093/nar/18.17.4993 PMC332103

[14] Engelhorn M, Boccard F, Murtin C, Prentki P, Geiselmann J (1995) In vivo interaction of the Escherichia coli integration host factor with its specific binding sites. Nucleic Acids Res 23: 2959–2965. 7567442

[15] Ussery D, Larsen TS, Wilkes KT, Friis C, Worning P, et al (2001) Genome organisation and chromatin structure in Escherichia coli. Biochimie 83: 201–212. 11278070 10.1016/s0300-9084(00)01225-6

[16] Wang S, Cosstick R, Gardner JF, Gumport RI (1995) The specific binding of Escherichia coli integration host factor involves both major and minor grooves of DNA. Biochemistry 34: 13082–13090. 7548068 10.1021/bi00040a020

[17] Yang SW, Nash HA (1995) Comparison of protein binding to DNA in vivo and in vitro: defining an effective intracellular target. EMBO J 14: 6292–6300. 8557048 10.1002/j.1460-2075.1995.tb00319.x PMC394753

[18] Murtin C, Engelhorn M, Geiselmann J, Boccard F (1998) A quantitative UV laser footprinting analysis of the interaction of IHF with specific binding sites: re-evaluation of the effective concentration of IHF in the cell. J Mol Biol 284: 949–961. 9837718 10.1006/jmbi.1998.2256

[19] Craig NL, Nash HA (1984) E. coli integration host factor binds to specific sites in DNA. Cell 39: 707–716. 6096022 10.1016/0092-8674(84)90478-1

[20] Nienhuis AW, Bunn HF, Turner PH, Gopal TV, Nash WG, et al (1985) Expression of the human c-fms proto-oncogene in hematopoietic cells and its deletion in the 5q- syndrome. Cell 42: 421–428. 4028159 10.1016/0092-8674(85)90099-6

[21] Sugimura S, Crothers DM (2006) Stepwise binding and bending of DNA by Escherichia coli integration host factor. Proc Natl Acad Sci U S A 103: 18510–18514. 17116862 10.1073/pnas.0608337103 PMC1654134

[22] Vivas P, Kuznetsov SV, Ansari A (2008) New insights into the transition pathway from nonspecific to specific complex of DNA with Escherichia coli integration host factor. J Phys Chem B 112: 5997–6007. 18461910 10.1021/jp076042s

[23] Whitchurch CB, Tolker-Nielsen T, Ragas PC, Mattick JS (2002) Extracellular DNA required for bacterial biofilm formation. Science 295: 1487. 11859186 10.1126/science.295.5559.1487

[24] Steinberger RE, Holden PA (2005) Extracellular DNA in single- and multiple-species unsaturated biofilms. Appl Environ Microbiol 71: 5404–5410. 16151131 10.1128/AEM.71.9.5404-5410.2005 PMC1214645

[25] Goodman SD, Obergfell KP, Jurcisek JA, Novotny LA, Downey JS, et al (2011) Biofilms can be dispersed by focusing the immune system on a common family of bacterial nucleoid-associated proteins. Mucosal Immunol 4: 625–637. 21716265 10.1038/mi.2011.27

[26] van Noort J, Verbrugge S, Goosen N, Dekker C, Dame RT (2004) Dual architectural roles of HU: formation of flexible hinges and rigid filaments. Proc Natl Acad Sci U S A 101: 6969–6974. 15118104 10.1073/pnas.0308230101 PMC406450

[27] Xiao B, Johnson RC, Marko JF (2010) Modulation of HU-DNA interactions by salt concentration and applied force. Nucleic Acids Res 38: 6176–6185. 20497998 10.1093/nar/gkq435 PMC2952867

[28] Schnurr B, Vorgias C, Stavans J (2006) Compaction and supercoiling of single, long DNA molecules by HU protein. Biophys Rev Lett 1: 29–44.

[29] Holbrook JA, Tsodikov OV, Saecker RM, Record MT Jr (2001) Specific and non-specific interactions of integration host factor with DNA: thermodynamic evidence for disruption of multiple IHF surface salt-bridges coupled to DNA binding. J Mol Biol 310: 379–401. 11428896 10.1006/jmbi.2001.4768

[30] Vander Meulen KA, Saecker RM, Record MT Jr (2008) Formation of a wrapped DNA-protein interface: experimental characterization and analysis of the large contributions of ions and water to the thermodynamics of binding IHF to H’ DNA. J Mol Biol 377: 9–27. 18237740 10.1016/j.jmb.2007.11.104 PMC2336898

[31] Ali BM, Amit R, Braslavsky I, Oppenheim AB, Gileadi O, et al (2001) Compaction of single DNA molecules induced by binding of integration host factor (IHF). Proc Natl Acad Sci U S A 98: 10658–10663. 11535804 10.1073/pnas.181029198 PMC58522

[32] Yan J, Marko JF (2003) Effects of DNA-distorting proteins on DNA elastic response. Phys Rev E Stat Nonlin Soft Matter Phys 68: 011905. 12935174 10.1103/PhysRevE.68.011905

[33] Yan J, Kawamura R, Marko JF (2005) Statistics of loop formation along double helix DNAs. Phys Rev E Stat Nonlin Soft Matter Phys 71: 061905. 16089763 10.1103/PhysRevE.71.061905

[34] Vivas P, Velmurugu Y, Kuznetsov SV, Rice PA, Ansari A (2012) Mapping the Transition State for DNA Bending by IHF. J Mol Biol 418: 300–315. 22370561 10.1016/j.jmb.2012.02.028

[35] Liu Y, Chen H, Kenney LJ, Yan J (2010) A divalent switch drives H-NS/DNA-binding conformations between stiffening and bridging modes. Genes Dev 24: 339–344. 20159954 10.1101/gad.1883510 PMC2816733

[36] Lim CJ, Whang YR, Kenney LJ, Yan J (2012) Gene silencing H-NS paralogue StpA forms a rigid protein filament along DNA that blocks DNA accessibility. Nucleic Acids Res 40: 3316–3328. 22187157 10.1093/nar/gkr1247 PMC3333869

[37] Winardhi RS, Fu W, Castang S, Li Y, Dove SL, et al.. (2012) Higher order oligomerization is required for H-NS family member MvaT to form gene-silencing nucleoprotein filament. Nucleic Acids Res Advance Access published July 13, 2012. 10.1093/nar/gks669 PMC3467065 22798496

[38] Nash HA, Robertson CA, Flamm E, Weisberg RA, Miller HI (1987) Overproduction of Escherichia coli integration host factor, a protein with nonidentical subunits. J Bacteriol 169: 4124–4127. 3305480 10.1128/jb.169.9.4124-4127.1987 PMC213718

[39] Yan J, Skoko D, Marko J (2004) Near-field-magnetic-tweezer manipulation of single DNA molecules. Phys Rev E 70: 011905. 10.1103/PhysRevE.70.011905 15324086

[40] Strick TR, Allemand JF, Bensimon D, Bensimon A, Croquette V (1996) The elasticity of a single supercoiled DNA molecule. Science 271: 1835–1837. 8596951 10.1126/science.271.5257.1835

[41] Marko JF, Siggia ED (1995) Stretching DNA. Macromolecules 28: 8759–8770.

[42] Fu H, Freedman BS, Lim CT, Heald R, Yan J (2011) Atomic force microscope imaging of chromatin assembled in Xenopus laevis egg extract. Chromosoma 120: 245–254. 21369955 10.1007/s00412-010-0307-4 PMC3464096

[43] Wang H, Bash R, Yodh JG, Hager GL, Lohr D, et al (2002) Glutaraldehyde modified mica: a new surface for atomic force microscopy of chromatin. Biophys J 83: 3619–3625. 12496129 10.1016/S0006-3495(02)75362-9 PMC1302437

[44] Bao Q, Chen H, Liu Y, Yan J, Droge P, et al (2007) A divalent metal-mediated switch controlling protein-induced DNA bending. J Mol Biol 367: 731–740. 17276457 10.1016/j.jmb.2006.09.082

[45] Chen H, Yan J (2008) Effects of kink and flexible hinge defects on mechanical responses of short double-stranded DNA molecules. Phys Rev E Stat Nonlin Soft Matter Phys 77: 041907. 18517656 10.1103/PhysRevE.77.041907

[46] Smith SB, Finzi L, Bustamante C (1992) Direct mechanical measurements of the elasticity of single DNA molecules by using magnetic beads. Science 258: 1122–1126. 1439819 10.1126/science.1439819

[47] Sagi D, Friedman N, Vorgias C, Oppenheim AB, Stavans J (2004) Modulation of DNA conformations through the formation of alternative high-order HU-DNA complexes. J Mol Biol 341: 419–428. 15276833 10.1016/j.jmb.2004.06.023

[48] Lusk JE, Williams RJ, Kennedy EP (1968) Magnesium and the growth of Escherichia coli. J Biol Chem 243: 2618–2624. 4968384

[49] Paymaster NJ (1976) Magnesium-Metabolism - Brief Review. Ann R Coll Surg Engl 58: 309–314. 942168 PMC2493703

[50] Walthers D, Li Y, Liu YJ, Anand G, Yan J, et al (2011) Salmonella enterica Response Regulator SsrB Relieves H-NS Silencing by Displacing H-NS Bound in Polymerization Mode and Directly Activates Transcription. J Biol Chem 286: 1895–1902. 21059643 10.1074/jbc.M110.164962 PMC3023485

[51] Frenkiel-Krispin D, Levin-Zaidman S, Shimoni E, Wolf SG, Wachtel EJ, et al (2001) Regulated phase transitions of bacterial chromatin: a non-enzymatic pathway for generic DNA protection. EMBO J 20: 1184–1191. 11230141 10.1093/emboj/20.5.1184 PMC145506

[52] Fu WB, Wang XL, Zhang XH, Ran SY, Yan J, et al (2006) Compaction dynamics of single DNA molecules under tension. J Am Chem Soc 128: 15040–15041. 17117826 10.1021/ja064305a

[53] Sarkar T, Petrov AS, Vitko JR, Santai CT, Harvey SC, et al (2009) Integration host factor (IHF) dictates the structure of polyamine-DNA condensates: implications for the role of IHF in the compaction of bacterial chromatin. Biochemistry 48: 667–675. 19132923 10.1021/bi8019965

[54] Skoko D, Wong B, Johnson RC, Marko JF (2004) Micromechanical analysis of the binding of DNA-bending proteins HMGB1, NHP6A, and HU reveals their ability to form highly stable DNA-protein complexes. Biochemistry 43: 13867–13874. 15504049 10.1021/bi048428o

[55] McCauley M, Hardwidge PR, Maher LJ III, Williams MC (2005) Dual binding modes for an HMG domain from human HMGB2 on DNA. Biophys J 89: 353–364. 15833996 10.1529/biophysj.104.052068 PMC1366535

[56] Skoko D, Yan J, Johnson RC, Marko JF (2005) Low-force DNA condensation and discontinuous high-force decondensation reveal a loop-stabilizing function of the protein Fis. Phys Rev Lett 95: 208101. 16384101 10.1103/PhysRevLett.95.208101

[57] Skoko D, Yoo D, Bai H, Schnurr B, Yan J, et al (2006) Mechanism of chromosome compaction and looping by the Escherichia coli nucleoid protein Fis. J Mol Biol 364: 777–798. 17045294 10.1016/j.jmb.2006.09.043 PMC1988847

[58] Azam TA, Hiraga S, Ishihama A (2000) Two types of localization of the DNA-binding proteins within the Escherichia coli nucleoid. Genes Cells 5: 613–626. 10947847 10.1046/j.1365-2443.2000.00350.x

[59] Hildebrandt ER, Cozzarelli NR (1995) Comparison of recombination in vitro and in E. coli cells: measure of the effective concentration of DNA in vivo. Cell 81: 331–340. 7736586 10.1016/0092-8674(95)90386-0

[60] Wang W, Li GW, Chen C, Xie XS, Zhuang X (2011) Chromosome organization by a nucleoid-associated protein in live bacteria. Science 333: 1445–1449. 21903814 10.1126/science.1204697 PMC3329943

[61] Flemming HC, Wingender J (2010) The biofilm matrix. Nat Rev Microbiol 8: 623–633. 20676145 10.1038/nrmicro2415

[62] Stinson MW, Bergey EJ (1982) Isolation of heart- and kidney-binding protein from group A streptococci. Infect Immun 35: 335–342. 7033140 10.1128/iai.35.1.335-342.1982 PMC351034

[63] Korstgens V, Flemming HC, Wingender J, Borchard W (2001) Influence of calcium ions on the mechanical properties of a model biofilm of mucoid Pseudomonas aeruginosa. Water Sci Technol 43: 49–57. 11381972

