# Supplementary figures and images for: Physical Organization of DNA by Multiple Non-Specific DNA-Binding Modes of Integration Host Factor (IHF)
Source: PLoS One. 2012 Nov 14;7(11):e49885. doi: 10.1371/journal.pone.0049885 (PMC3498176; doi:10.1371/journal.pone.0049885)

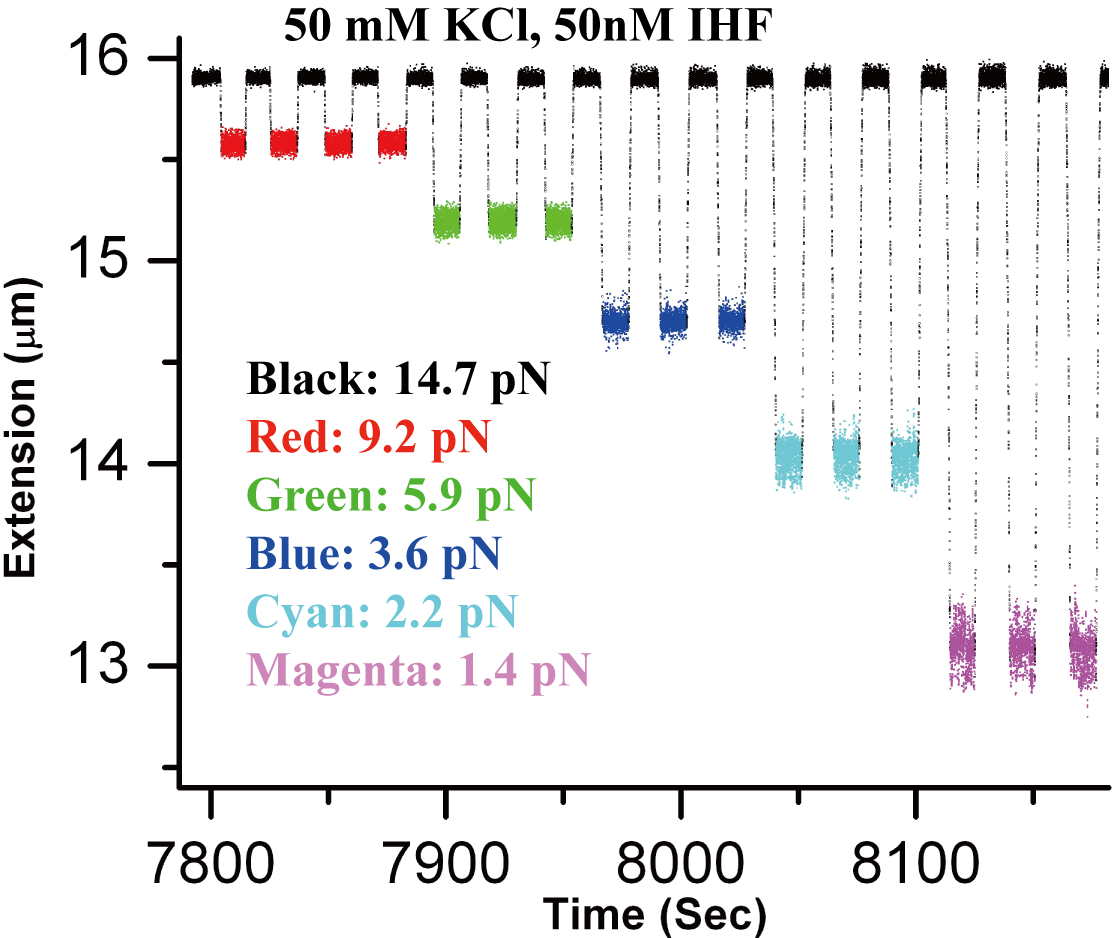

Supplement: Figure S1 — λ-DNA extension time-course in a force-jumping experiment at 50 nM IHF in 50 mM KCl. Black indicates the highest force (14.7 pN). Forces of lower values are indicated by different colors. (TIF) [file pone.0049885.s001.tif]

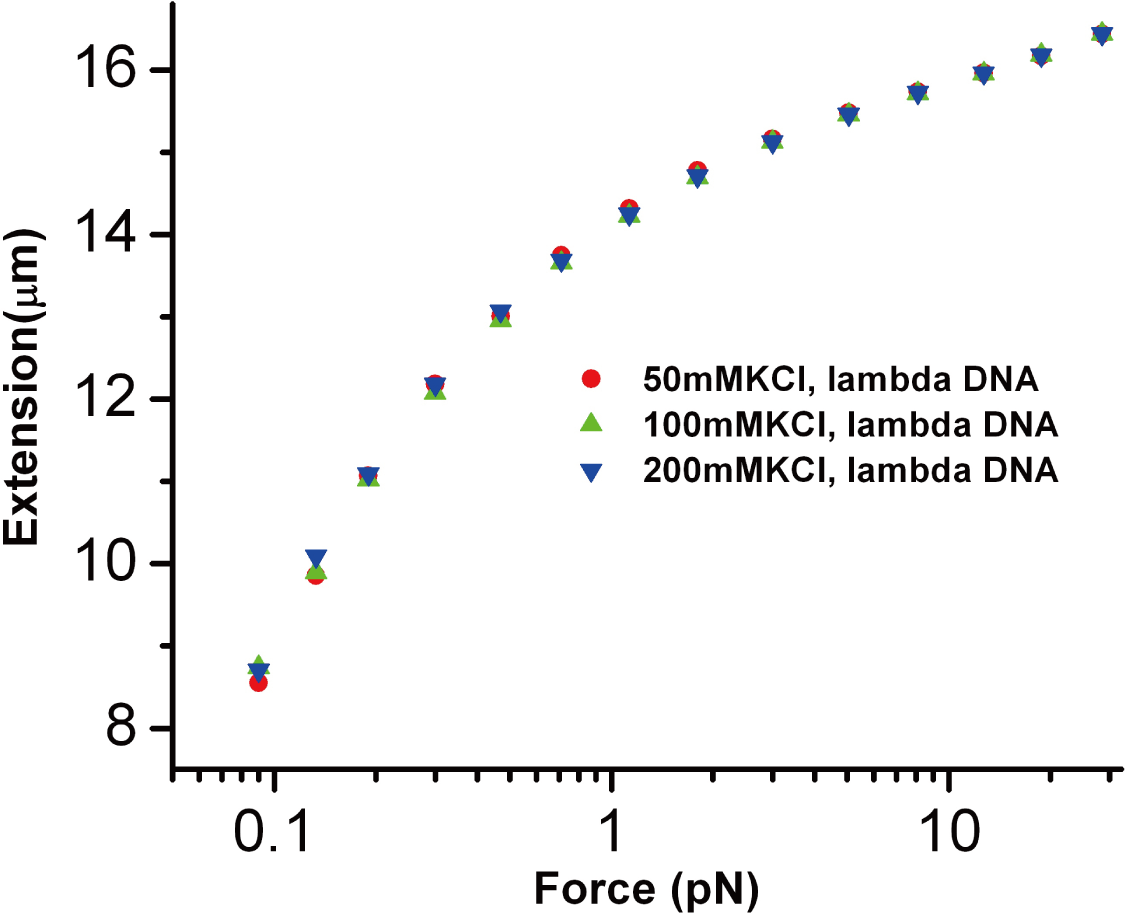

Supplement: Figure S2 — Force-extension curves of λ-DNA in 50–200 mM KCl and pH 7.4 (10 mM Tris). These show that the force-response of DNA is almost identical in the whole KCl concentration range. (TIF) [file pone.0049885.s002.tif]

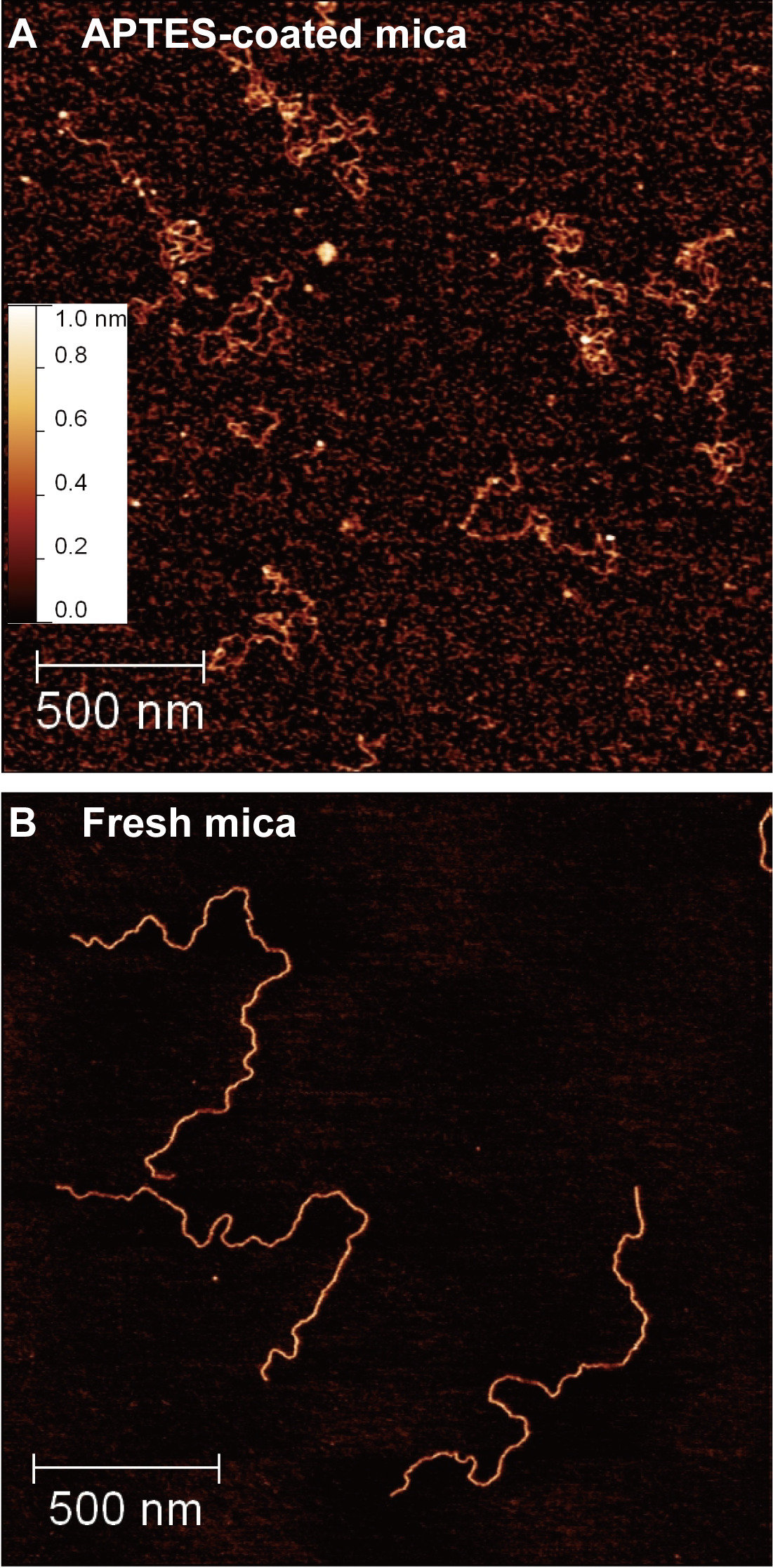

Supplement: Figure S3 — AFM imaging of naked Φx174 DNA on APTES-coated mica and freshly cleaved mica. (A) Naked DNA in 200 mM KCl on APTES-modified mica. (B) Naked DNA in 10 mM MgCl2 (divalent salt bridging) on Fresh-mica surface. (TIF) [file pone.0049885.s003.tif]

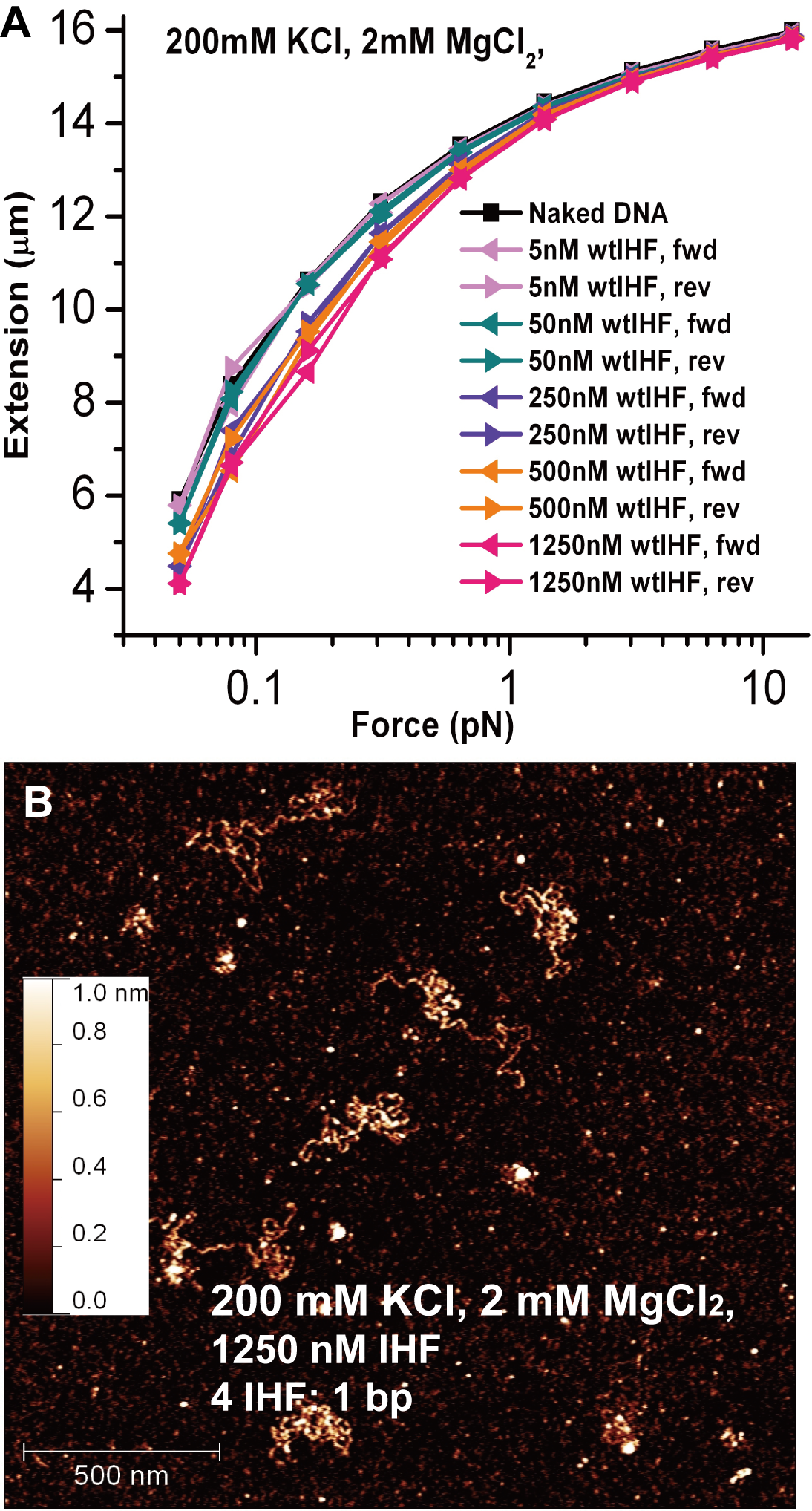

Supplement: Figure S4 — IHF-DNA interaction in 200 mM KCl in the presence of magnesium. (A) Effects of magnesium on DNA conformations in 200 mM KCl. Force-extension curves in force-decreasing and force-increasing scans of λ-DNA at the indicated IHF concentrations, which are similar to those obtained in 200 mM KCl in the absence of magnesium (Figure 1B). (B) AFM imaging of DNA molecules complexed with 1250 nM IHF in 200 mM KCl in the present of 2 mM MgCl2. (TIF) [file pone.0049885.s004.tif]

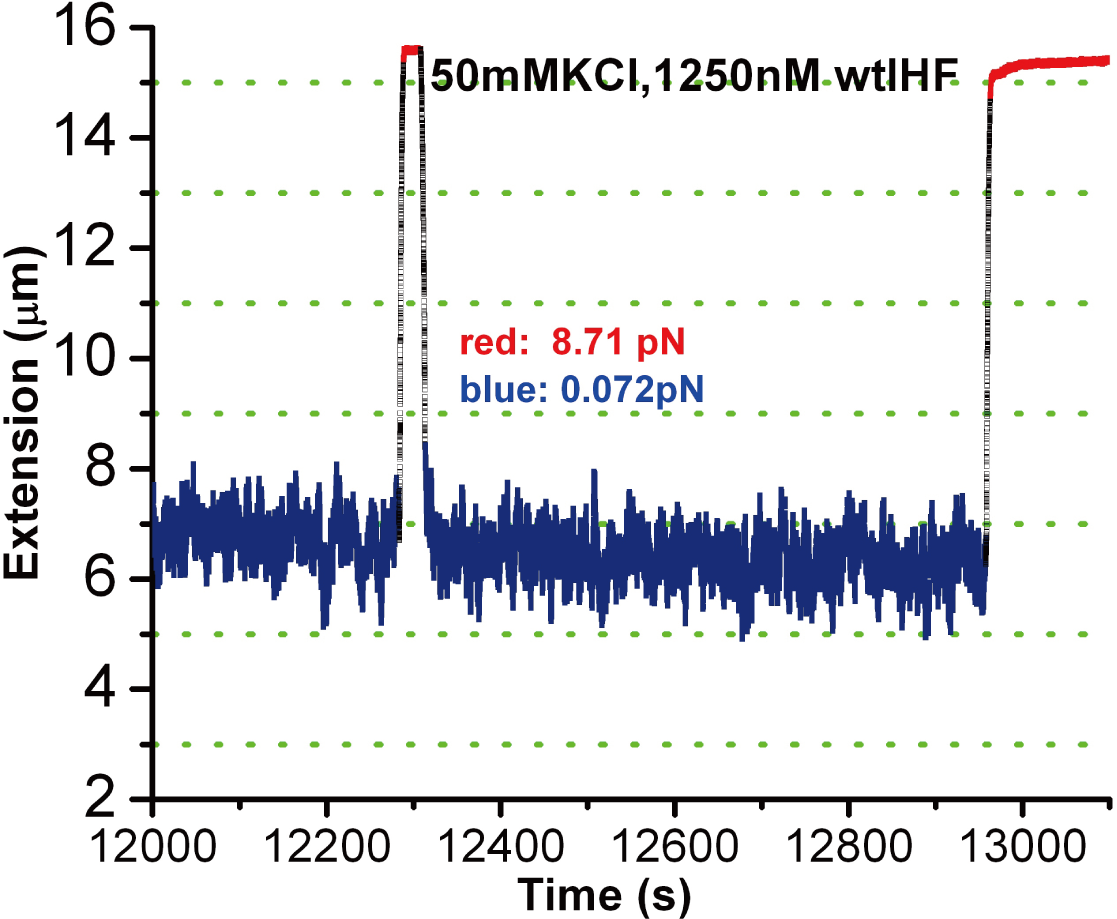

Supplement: Figure S5 — Folding time course of λ-DNA with 1250 nM IHF in 50 mM KCl solution. The compaction without magnesium is much slower (blue curve), even at the lowest force ∼0.07 pN, compared to that in the similar 50 mM KCl solution with magnesium (Figure 3B). Moreover, the compaction is not as stable as that with magnesium, as it can be easily unfolded under at ∼8.7 pN (red curve). The green dot grids are used as a comparison criterion for the DNA extension reduction. (TIF) [file pone.0049885.s005.tif]
