# Supplementary material for: Physical Organization of DNA by Multiple Non-Specific DNA-Binding Modes of Integration Host Factor (IHF)
Source: PLoS One. 2012 Nov 14;7(11):e49885. doi: 10.1371/journal.pone.0049885 (PMC3498176; doi:10.1371/journal.pone.0049885)
Supplement: Methods S1 — Supplementary Methods. (A) Quick force jumping method (B) Simulation details. (DOC) [file pone.0049885.s006.doc]

**Methods S1**

**Physical organization of DNA by multiple non-specific DNA-binding modes of Integration Host Factor (IHF)**

Jie Lin, Hu Chen, Peter Dröge, and Jie Yan

1. **Quick force jumping method**

To distinguish immediate DNA bending from the sharper DNA bending with slower kinetics or DNA folding into higher order structures, the interference from these slower kinetics can be minimized by reducing the time at which DNA is held at small force values using a quick force jumping method. Supporting Figure S1 is the DNA extension time course in a force-jumping experiment at 50 mM KCl and 50 nM IHF. First, the DNA is held at >10 pN (~14.7 pN, black curve) to prevent folding while IHF solution is added to the reaction channel. Next, the force is jumped to a lower value (colored curves) and DNA extension is recorded by holding the DNA for ~ 10 seconds. The force is then brought back to the highest force (black curve) to ensure that extension returns to the original value of the naked DNA. Repeating the process at each force for at least three times, the average extension and the standard deviation are obtained. The force-extension curve of DNA is obtained by operating the same procedure for a series of lower force values. Through this quick force jumping method, the slower DNA bending or folding kinetics that occur at a lower force value will not accumulate at the next lower force value; thus, its interference is reduced. The force-jumping method was used to obtain data in Figure 1E.

1. **Simulation details**

The DNA is modeled as a worm-like chain polymer with a bending persistence length of A=50 nm . In the simulation, the DNA is discretized to a series of N short segments, each of length b=1 nm. The bending of two adjacent segments costs an energy of , where $\hat{t}_{i}$ are tangent vectors describing segment orientations. This discrete model is equivalent to the continuum WLC model when b<<A, and a=A/b. When IHF binding causes a bending angle, the corresponding bending energy is modified to be: , where $\theta_{*}$ is the preferred bending angle at the protein bound vertex connecting two adjacent segments . The total conformational energy of the DNA is the sum of all the vertex energies: .

In the simulation, the IHF-induced kinks are uniformly distributed along DNA. The spacing between two adjacent kinks describes the occupancy density of IHF on the DNA. The resulting force-extension curve of DNA is calculated by the transfer-matrix method developed recently . Using this method, the values of the spacing between the adjacent IHF-induced bends and the bending angle that can decrease extension from naked DNA at 0.1 pN by 50% are calculated and plotted in Figure 1H.

**REFERENCES OF METHODS S1**

1. Smith SB, Finzi L, Bustamante C (1992) Direct mechanical measurements of the elasticity of single DNA molecules by using magnetic beads. Science 258: 1122-1126.

2. Marko JF, Siggia ED (1995) Stretching DNA. Macromolecules 28: 8759-8770.

3. Yan J, Marko JF (2003) Effects of DNA-distorting proteins on DNA elastic response. Phys Rev E Stat Nonlin Soft Matter Phys 68: 011905.

4. Yan J, Kawamura R, Marko JF (2005) Statistics of loop formation along double helix DNAs. Phys Rev E Stat Nonlin Soft Matter Phys 71: 061905.
